# Rabenchromenone and Rabenzophenone, Phytotoxic Tetrasubstituted Chromenone and Hexasubstituted Benzophenone Constituents Produced by the Oak-Decline-Associated Fungus *Fimetariella rabenhorstii*

**DOI:** 10.1021/acs.jnatprod.9b01017

**Published:** 2020-01-22

**Authors:** Samaneh Bashiri, Jafar Abdollahzadeh, Roberta Di Lecce, Daniela Alioto, Marcin Górecki, Gennaro Pescitelli, Marco Masi, Antonio Evidente

**Affiliations:** † Department of Plant Protection, Faculty of Agriculture, University of Kurdistan, Pasdaran Street, Post Office Box 416, Sanandaj, Iran; ‡ Dipartimento di Scienze Chimiche, Università di Napoli Federico II, Complesso Universitario Monte Sant’Angelo, Via Cintia 4, 80126 Napoli, Italy; § Dipartimento di Agraria, Università degli Studi di Napoli Federico II, Via Università 100, 80055 Portici, Italy; ∥ Dipartimento di Chimica e Chimica Industriale, Università di Pisa, Via Moruzzi 13, 56124 Pisa, Italy; ⊥ Institute of Organic Chemistry, Polish Academy of Sciences, Kasprzaka 44/52 Street, 01-224 Warsaw, Poland

## Abstract

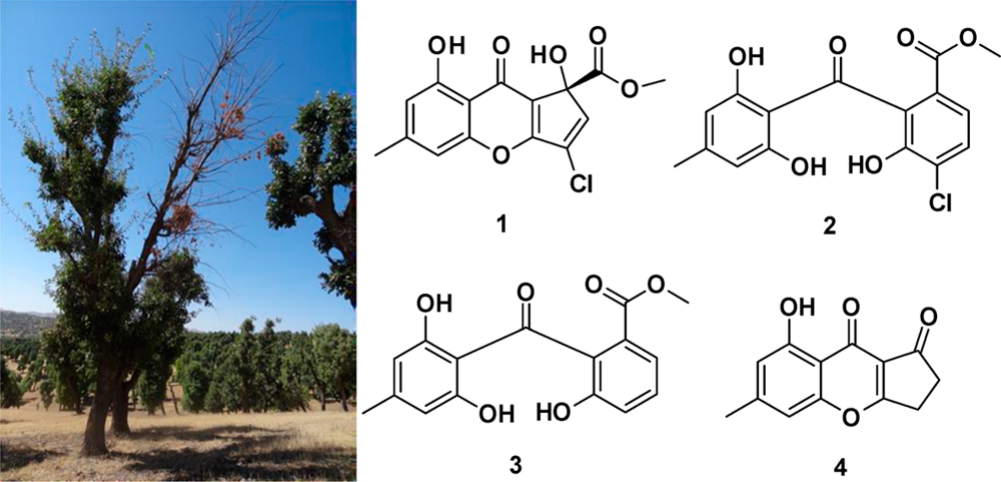

A new phytotoxic tetrasubstituted chromen-4-one (**1**) and a new hexasubstituted benzophenone (**2**), named rabenchromenone and rabenzophenone, respectively, were isolated from the culture filtrates of *Fimetariella rabenhorstii*, an oak-decline-associated fungus in Iran. Rabenchromenone and rabenzophenone, isolated together with known moniliphenone (**3**) and coniochaetone A (**4**), were characterized as methyl 3-chloro-1,8-dihydroxy-6-methyl-9-oxo-1,9-dihydrocyclopenta[*b*]chromene-1-carboxylate and methyl 4-chloro-2-(2,6-dihydroxy-4-methylbenzoyl)-3-hydroxybenzoate, respectively, by spectroscopic methods (primarily nuclear magnetic resonance and high-resolution electrospray ionization mass spectrometry). The *R* absolute configuration at C-1 of rabenchromenone was determined by quantum chemical calculations and electronic circular dichroism experiments. All metabolites (**1**–**4**) were tested by leaf puncture on tomato and oak plants. All compounds were active in this assay by causing in both plants a necrosis diameter in the range of 0.2–0.7 cm. Specifically, rabenzophenone (**2**) was found to be the most phytotoxic compound in both plants.

There are several fungi associated with oak trees, including saprobes, endophytes, pathogens, and mutualistic mycorrhizal fungi, that ensure a given tree’s good health and, ultimately, survival.^1^ Endophytes are organisms, mostly bacteria and fungi, that can colonize internal plant tissues without causing damage to the host, but latent opportunistic pathogens can also be included in this group.^2−7^ Some endophytic fungi may exist as latent or inactive pathogens but become active or may change their mode of nutrition and function during their life cycle when their host plants are stressed or under certain environmental conditions.^8−10^ Endophytic fungi colonize a relatively unexplored ecological habitat and are a rich source of new and bioactive metabolites.^1^ On the other hand, generally two different lifestyles or nutritional modes, biotrophic and necrotrophic, have been recognized in plant pathogenic fungi. Phytotoxic metabolites are a major component of the weaponry system of necrotrophs that act as virulence or pathogenicity factors in host–pathogen interactions and in the infection process.^11^ Thus, isolation and characterization of these phytotoxins are the first steps to understand their role in the phytopathogenic process and the induction of symptoms for disease management.

*Fimetariella rabenhorstii* (Niessl) N. Lundq. (Ascomycota, Sordariales, Lasiosphaeriaceae), recently isolated as endophytic fungus associated with *Aquilaria sinensis* in mainland China,^12^ was detected for the first time from oak trees (*Quercus brantii*), showing decline and wood necrosis symptoms from the Zagros forest, in the west of Iran (Figure 1). Considering the current limited knowledge on the secondary metabolites produced by this pathogen, the main objective of this research was to isolate and characterize the bioactive compounds produced *in vitro* by *F. rabenhorstii* and to evaluate their phytotoxicity.

**Figure 1:**
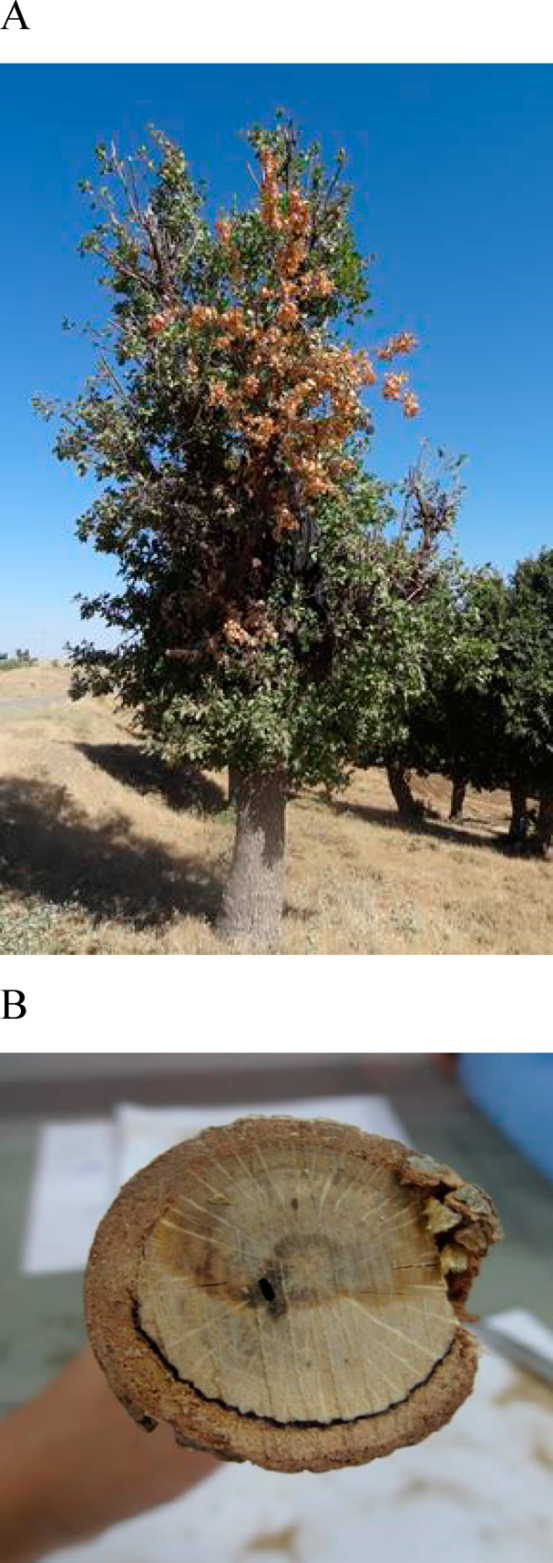
Symptoms of *Q. brantii* tree affected by *F. rabenhorstii*: (A) oak decline and (B) necrosis in a trunk cross-section.

The availability of phytotoxins could allow for the development of rapid and specific methods for disease diagnoses^13^ and the investigation of other biological activities for potential application in agriculture (as natural herbicides, fungicides, bacteriocides, insecticides, etc.)^14^ and medicine (as antitumor and antimosquito agents, etc.).^15−18^

This manuscript describes the isolation of four metabolites from the culture filtrates of *F. rabenhorstii* and their chemical and biological characterization.

## Results and Discussion

**Figure 2:**
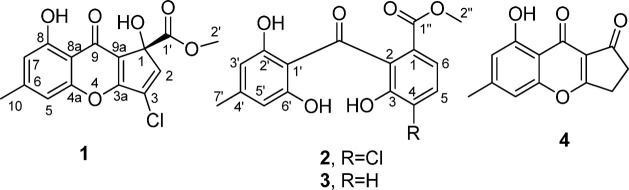
Structures of rabenchromenone, rabezophenone, moniliphenone, and coniochaetone A (**1**–**4**).

The EtOAc extract of *F. rabenhorstii* culture filtrates, showing a strong phytotoxicity, was fractionated by thin-layer chromatography (TLC) and column chromatography (CC), as detailed in the Experimental Section. The purification process yielded four metabolites (**1**–**4**; Figure 2), a new tetrasubstituted chromenone and a new hexasubstituted benzophenone, named rabenchromenone and rabenzophenone, respectively (**1** and **2**; Figure 2) and two already known compounds, which were identified as moniliphenone and coniochaetone A (**3** and **4**; Figure 2). The last two metabolites were identified by comparison of their specific optical properties and spectroscopic data [^1^H and ^13^C nuclear magnetic resonance (NMR) and electrospray ionization mass spectrometry (ESIMS)] to those previously reported for compounds **3**(19) and **4**.^20,21^ Moniliphenone (**3**) was isolated for the first time from *Monilia fructicola* as an intermediate of chloromonilicin biosynthesis.^19^ Successively, compound **3** was isolated from several other terrestrial and marine fungi, such as *Coniochaeta* sp.,^22^*Hypocreales* sp.,^23^*Aspergillus sydowii*,^24^*Leptosphaeria* sp.,^25^ and *Penicillium* spp.^26^ Coniochaetone A (**4**) was isolated for the first time together with coniochaetone B, an antifungal metabolite from *Coniochaeta saccardoi*.^20^ Compound **4** was also produced by *Fimetariella* sp. (S207) obtained from a sample of *Ophiocordyceps sinensis* collected in the People’s Republic of China together with the new cheniochaetones E–I and coniochaetone B.^27^

Preliminary ^1^H and ^13^C NMR investigation of rabenchromenone (**1**) and ranbenzophenone (**2**) suggested that they belong to two different subgroups of natural compounds as 4*H*-chromen-4-one and benzoate derivatives. Rabenchromenone has a molecular formula of C_15_H_11_ClO_6_, as determined from its high-resolution electrospray ionization mass spectrometry (HRESIMS), consistent with 10 indices of hydrogen deficiency. Its infrared (IR) spectrum showed bands typical for aromatic, hydroxy, olefinic, and carbonyl groups,^28^ while its ultraviolet (UV) spectrum showed absorption maxima as a result of the presence of extended conjugated chromophores.^29^ These results were in agreement with the initial investigation of its ^1^H and ^13^C NMR data (Table 1). In particular, its ^1^H NMR and correlation spectroscopy (COSY) spectra^29^ showed a singlet typical of a hydrogen-bonded phenolic hydroxy group at δ 12.11, two broad singlets typical of two *meta*-coupled (*J* < 1 Hz) aromatic protons at δ 6.89 (H-7) and 6.70 (H-5), and two other singlets typical of a vinyl methyl (Me-10) and methoxy group at δ 2.43 and 3.79, respectively.^29^ Its ^13^C NMR spectrum (Table 1) showed the presence of signals for two carbonyls, one of a conjugated ketone and the other one of an ester group, for three sp^2^ methines, for a vinyl methyl, for a methoxy, for seven quaternary sp^2^ carbons, three of which appeared to be oxygenated, and for one quaternary sp^3^ hydroxylated carbon. Hydrogenated carbons were assigned by the couplings observed in the heteronuclear single-quantum correlation (HSQC) spectrum^30^ (Table 1), and thus, the signals at δ 139.2, 113.7, 108.2, 54.1, and 22.2 were assigned to C-2, C-7, C-5, the methoxy group, and methyl (Me-10) at C-6. Carbonyl signals at δ 176.4 and 170.5 were assigned to C-9 and C-1′, also on the basis of the coupling between C-1′ and the methoxy group observed in the heteronuclear multiple-bond correlation (HMBC) spectrum^30^ (Table 1). The remaining quaternary carbons were assigned considering the other correlations observed in the same spectrum, in particular, C-1, C-3, C-3a, and C-9a coupled with H-2, C-4a and C-8a coupled with H-7, and C-6 with H-10. Thus, the signals resonating at δ 164.7, 156.3, 147.1, 127.1, 119.3, 109.3, and 79.6 were assigned to C-3a, C-4a, C-6, C-3, C-9a, C-8a, and C-1. The remaining quaternary signal at δ 161.0 was assigned to carbon C-8 linked to the phenolic hydroxy group.^31^ The couplings between C-10 and H-5 and H-7 allowed the benzyl methyl (Me-10) to be positioned at C-6 and, thus, the chlorine atom at C-3. Therefore, the chemical shifts of all of the protons and the corresponding carbons were assigned and reported in Table 1, and compound **1** was formulated as methyl 3-chloro-1,8-dihydroxy-6-methyl-9-oxo-1,9-dihydrocyclopenta[*b*]chromene-1-carboxylate.

**Table 1:** ^1^H and ^13^C NMR Data of Rabenchromenone (**1**)^a^^,^^b^

number	δ_C_^c^	δ_H_ (*J* in Hz)	HMBC
1	79.6 s		H-2
2	139.2 d	6.65 s	
3	127.1 s		H-2
3a	164.7 s		H-2
4a	156.3 s		H-7
5	108.2 d	6.70 br s	H_3_-10
6	147.1 s		H_3_-10
7	113.7 d	6.89 br s	H_3_-10
8	161.0 s		
8a	109.3 s		H-7
9	176.4 s		
9a	119.3 s		H-2
10	22.2 q	2.43 s	H-5, H-7
1′	170.5 s		H_3_-2′
2′	54.1 q	3.79 s	
OH		12.11 s	

a The chemical shifts are in δ values (ppm) from tetramethylsilane (TMS).

b Two-dimensional (2D) ^1^H, ^1^H (COSY) and ^13^C, ^1^H (HSQC) NMR experiments delineated the correlations of all of the protons and the corresponding carbons.

c Multiplicities were assigned by the distortionless enhancement by polarization transfer (DEPT) spectrum.

The structure attributed to compound **1** was supported by the data of its HRESIMS spectrum, which showed the dimer potassium [2M + K]^+^ and sodium [2M + Na]^+^ adducts, the sodium adduct [M + Na]^+^, and the protonated [M + H]^+^ ions at *m*/*z* 683.0139, 667.0417, 345.0155, and 323.0334, respectively. The same spectrum showed the typical isotopic peaks of ^37^Cl at *m*/*z* 685.0119, 669.0384, 347.0126, and 325.0113.

**Figure 3:**
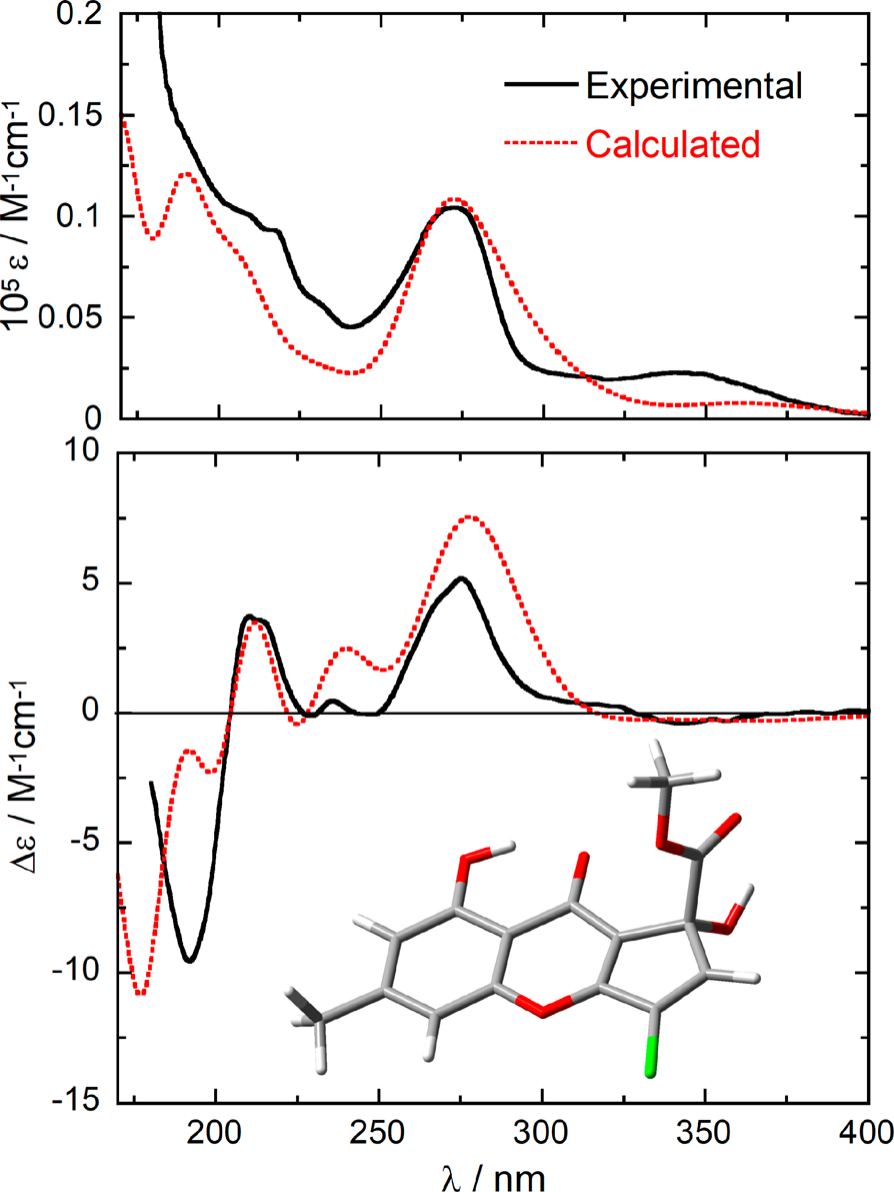
(Top) Ultraviolet–visible (UV–vis) absorption and (bottom) ECD spectra of compound **1** measured in CH_3_CN (solid lines, 4.6 mM, 0.01 cm cell) compared to spectra calculated for (*R*)-**1** at the TD-B3LYP/def2-TZVP/PCM//ωB97X-D/6-311+G(d,p)/SMD level as the Boltzmann average of two conformers at 300 K (dotted lines). Calculated spectra were obtained as sums of Gaussian bands with 0.3 eV exponential half width, blue-shifted by 5 nm. The ECD spectrum was scaled by a factor of 0.3. The inset structure is the lowest energy conformer of compound **1**.

The absolute configuration of rabenchromenone (**1**) was determined by application of electronic circular dichroism (ECD) spectroscopy supported by quantum mechanical calculations.^32,33^ Compound **1** has a rigid structure, and only two possible conformers were found by a molecular mechanics conformational search and density functional theory (DFT) geometry optimizations. The two conformers differ in the orientation of the ester group and to a smaller extent the C1–OH group; in the most stable conformation (see Figure 3) the ester C=O points toward O–H. The absorption UV and ECD spectra were measured in acetonitrile and displayed several bands associated with the extended conjugated chromophore (Figure 3). The ECD spectrum calculated at the B3LYP/def2-TZVP/PCM level on the (*R*)-enantiomer of compound **1** matched in a satisfactorily manner the experimental spectrum in terms of band signs, positions and intensities (Figure 3); the results obtained with other functionals (see the Computational Methods) were also consistent. Thus, the absolute configuration of rabenchromenone was established as (*R*)-**1**.

Rabenzophenone (**2**) gave a molecular formula of C_16_H_13_ClO_6_ as determined from its HRESIMS, consistent with 10 indices of hydrogen deficiency. Its UV spectrum exhibited the absorption maxima for an extended conjugated system,^29^ while its IR spectrum showed bands typical of carbonyl, hydroxy, and aromatic groups.^28^ These findings are in agreement with its ^1^H and ^13^C NMR data (Table 2). In particular, the ^1^H and COSY NMR spectra showed the presence of two doublets (*J* = 8.3 Hz) at δ 7.56 (H-6) and 7.43 (H-5) and a broad singlet at δ 6.23 as a result of the overlapping of two aromatic *meta*-coupled protons (H-3′ and H-5′) typical of 1,2,4,6- and 1,2,3,4-tetrasubstituted benzene rings, respectively. This second ring, also from the signals observed in the ^13^C NMR spectrum, appeared symmetrically substituted. The ^1^H NMR spectrum also showed singlets for a benzyl methyl (Me-7′) and a methoxy group at δ 2.25 and 3.77, respectively.^29^ The ^13^C NMR spectrum showed the presence of two carbons typical of a ketone and an ester carbonyl (C-1″) at δ 197.3 and 166.3, respectively. In the HMBC spectrum (Table 2), the ketone and the ester carbonyl (C-1″) correlated with H-3′,5′ and with H-6 and the methoxy group, respectively. The ^13^C NMR spectrum also showed the presence of four protonated sp^2^ carbons that were assigned on the basis of the coupling observed in the HSQC spectrum (Table 2). In particular, the signals at δ 129.4, 123.2, and 109.9 were assigned to C-5, C-6, and C-3′,5′. Furthermore, the same spectrum showed the presence of eight sp^2^ quaternary carbons, three of which are oxygenated (C-2′,6′ and C-3) and two of them equivalent (C-2′,6′). These were assigned also by the couplings observed in the HMBC spectrum (Table 2) between C-1 and C-4 and H-5 and H-6, between C-2 and C-3 and H-5, between C-1 and C-2′,6′ and H-3′,5′, and between C-4′ and Me-7′. Thus, the signals at δ 160.9, 149.4, 148.2, 132.6, 128.0, 125.6, and 109.5 were assigned to C-2′,6′, C-4′, C-3, C-4, C-2, C-1, and C-1′.^31^ The chemical shifts were assigned to all carbons and the corresponding protons, as listed in Table 2, and compound **2** was determined as methyl 4-chloro-2-(2,6-dihydroxy-4-methylbenzoyl)-3-hydroxybenzoate.

**Table 2:** ^1^H and ^13^C NMR Data of Rabenzophenone (**2**)

number	δ_C_	δ_H_ (*J* in Hz)	HMBC
1	125.6 s		H-5, H-6
2	128.0 s		H-5
3	148.2 s		H-5
4	132.6 s		H-5, H-6
5	129.4 d	7.43 d (8.3)	H-6
6	123.2 d	7.56 d (8.3)	H-5
1′	109.5 s		H-3′,5′
2′,6′	160.9 s		H-3′,5′
3′,5′	109.9 d	6.23 br s	Me
4′	149.4 s		Me
CO	197.3 s		H-3′,5′
C-1″	166.3 s		H-6,OMe
Me-7′	22.5 q	2.25 s	H-3′,5′
OMe	53.1 q	3.77 s	

The structure assigned to rabenzophenone (**2**) was supported from the other couplings observed in the HMBC spectrum and its HRESIMS data. The latter showed a pseudo-molecular ion [M + H]^+^ and typical isotopic peaks of ^37^Cl at *m*/*z* 339.0458 and 337.0469. The significant fragment-stable acyl ion of the benzoate moiety observed at *m*/*z* 216.003 and 214.0039 was generated from the pseudo-molecular ion by the loss of a diphenoxy moiety.

Rabenzophenone (**2**) differs from moniliphenone (**3**), isolated from the same fungus as reported above, only by the presence of the chlorine atom at C-4.

The phytotoxic activity of metabolites **1**–**4** was estimated by a leaf puncture bioassay, on holm oak and tomato leaves (Table 3), at 1 mg/mL. All compounds were active in this assay, causing on both plants a necrosis diameter in the range between 0.2 and 0.5 cm. Specifically, compound **2** was the most phytotoxic compound on both plants and caused significant necrosis (0.7 and 0.5 cm on holm oak and tomato leaves, respectively).

**Table 3:** Phytotoxic Activity of Metabolites **1**–**4** Tested by Leaf Puncture at 1 mg/mL^a^

compound	holm oak (*Quercus ilex* L.)	tomato (*Lycopersicon esculentum* L.)
**1**	2	1
**2**	3	2
**3**	2	1
**4**	2	1

a Toxicity effects were expressed using a visual scale from 0 (no symptoms) to 4 (wide necrosis up to 1 cm in diameter).

In conclusion, four phytotoxic metabolites were isolated from *F. rabenhorstii* and two of them, named rabenchromenone (**1**) and rabenzophenone (**2**), were assigned as new 4*H*-benzochroman-4-one and new benzophenone derivatives. These are closely related to the already known moniliphenone (**3**) and coniochaetone (**4**) that were isolated for the first time from the same fungus and as phytotoxic metabolites.

4*H*-Benzochroman-4-ones are widely distributed as fungal metabolites,^34^ while cyclopentabenzopyran-4-ones are rare as natural compounds. However, coniochaetones A–I were previously reported as fungal metabolites.^20,21,27^ Benzophenones are a class of natural compounds including more than 300 substances with differently functionalized carbon skeletons. They have been reported from plants and fungi and exhibit several bioactive properties, including antifungal, antimicrobial, anti-HIV, antiviral, antioxidant, and cytotoxic.^35^

## Experimental Section

### General Experimental Procedures

IR spectra were recorded as glassy films on a PerkinElmer Spectrum 100 FTIR spectrometer. A JASCO V-530 spectrophotometer was used to record UV spectra in CH_3_CN solution. UV and ECD spectra of compound **1** were recorded, respectively, with a JASCO V-650 spectrophotomer and a JASCO J-715 spectropolarimeter, on a solution 4.6 mM in CH_3_CN and a quartz cell with 0.01 cm path length. ECD measurement parameters were the following: scan speed, 100 nm/min; time constant, 0.5 s; bandwidth, 1 nm; and accumulations, 8. An Anton Paar MCP 300 digital polarimeter was used to measure the optical rotation in MeOH. A Bruker instrument was used to record ^1^H, ^13^C and 2D NMR spectra at 400 and 100 MHz in CDCl_3_. The same solvent was also used as an internal standard. Carbon multiplicities were determined by DEPT spectra.^30^ DEPT, HSQC, COSY-45, and HMBC experiments^30^ were performed using Bruker microprograms. HRESIMS and ESIMS and liquid chromatography/mass spectrometry (LC/MS) analyses were performed using an Agilent 6230B LC/MS time-of-flight (TOF) system and 1260 Infinity high-performance liquid chromatography (HPLC). The HPLC separations were performed as previously described.^36^ CC was performed using silica gel (0.063–0.200 mm, Kieselgel 60, Merck), while analytical and preparative TLC were carried out on silica gel (0.25 and 0.5 mm, respectively, F_254_, Kieselgel 60) plates and reversed-phase (0.20 mm, F_254_, Kieselgel 60 RP-18, Merck) plates. The spots were visualized as previously described.^37^

### Fungal Strain

The isolate of *F. rabenhorstii* (SR84-1C) used in this study was isolated from stems of infected Iranian oak trees (*Q. brantii*) collected in a natural area in Kurdistan (Iran) and identified on the basis of internal transcribed spacer (ITS) sequence data. The pathogenicity of this fungus was confirmed on 2 year old oak trees in greenhouse conditions following Koch’s postulates.^38^ The pure culture was maintained on potato dextrose agar (PDA) and stored at 4 °C in the fungal collection of the Department of Plant Protection, University of Kurdistan, Sanandaj, Iran.

### Production, Extraction, and Purification

The fungus was grown under stationary conditions in 10 flasks containing 500 mL of modified Czapek–Dox medium (pH 6.8). The cultures were incubated at 25 °C in the dark for 30 days, after which the mycelium was removed by filtration through filter paper (Whatman No. 4). The filtrates (5.0 L) were lyophilized and stored at −20 °C until further processing. They were then dissolved in water (^1^/_10_ of the initial volume) and extracted with EtOAc (3 × 500 mL). The combined organic extracts were dehydrated by Na_2_SO_4_ and evaporated under reduced pressure, obtaining an orange–red oil residue (196 mg). This latter was fractioned by CC on silica gel (100 × 2 cm) and eluted with 1 L of CHCl_3_–MeOH (9:1), yielding 13 fractions. The residue (11.9 mg) of the third fraction was further purified by reversed-phase TLC eluted with MeOH–H_2_O (8:2), yielding an amorphous solid named rabenchromenone (**1**, 1.7 mg, 0.17 mg/L, and *R*_f_ of 0.36). The residue (10.0 mg) of the fifth fraction was further purified by TLC, eluted with CH_2_Cl_2_–iPrOH (95:5), yielding as an amorphous solid rabenzophenone (**2**, 1.1 mg, 0.11 mg/L, and *R*_f_ of 0.45). The residue (17.3 mg) of the sixth fraction was further purified by TLC and eluted with CHCl_3_–MeOH (9:1), yielding moniliphenone (**3**, 1.8 mg, 0.18 mg/L, and *R*_f_ of 0.31) as an amorphous solid. Finally, the residue (10.8 mg) of the fourth fraction was further purified by TLC, eluted with CHCl_3_–iPrOH (95:5), yielding coniochaetone A (**4**, 5.3 mg, 0.21 mg/L, and *R*_f_ of 0.56) as an amorphous solid.

#### Rabenchromenone (**1**):

[α]_D_^25^, +47 (*c* 0.06, MeOH); IR ν_max_, 3423, 1742, 1654, 1618, 1596, 1462 cm^–1^; UV λ_max_ (log ε), 341 (3.80), 272 (4.46) nm; ^1^H and ^13^C NMR data, see Table 1; HRESIMS (+), *m*/*z* 685.0119 and 683.0139 [2M + K]^+^, 669.0384 and 667.0417 [2M + Na]^+^, 347.0126 and 345.0155 [M + Na]^+^, 325.0113 and 323.0334 [M + H]^+^ (calcd for C_15_H_12_ClO_6_, 325.0293 and 323.0322).

#### Rabenzophenone (**2**):

IR ν_max_, 3346, 1733, 1635, 1588, 1465 cm^–1^; UV λ_max_ (log ε), 284 (2.50) nm; ^1^H and ^13^C NMR data, see Table 2; HRESIMS (+), *m*/*z* 339.0458 and 337.0469 [M + H]^+^ (calcd for C_16_H_14_ClO_6_, 339.0449 and 337.0479), 216.0013 and 214.0039 [C_9_H_7_ClO_4_ + H^+^]^+^.

#### Moniliphenone (**3**):

The ^1^H and ^13^C NMR spectra were very similar to those previously reported in the literature.^19^ HRESIMS (+), *m*/*z* 627.1488 [2M + Na]^+^, 325.0749 [M + Na]^+^, 303.0882 [M + H]^+^ (calcd for C_16_H_15_O_6_, 303.2867).

#### Coniochaetone A (**4**):

The ^1^H and ^13^C NMR spectra were very similar to those previously reported in the literature.^20^ HRESIMS (+), *m*/*z* 483.0391 [2M + Na]^+^, 253.4308 [M + Na]^+^, 231.5853 [M + H]^+^ (calcd for C_13_H_11_O_4_, 231.2240).

### Computational Methods

Molecular mechanics and preliminary DFT calculations were run with Spartan’18 (Wave function, Inc., Irvine, CA, U.S.A.), with standard parameters and convergence criteria. DFT and time-dependent density functional theory (TD-DFT) calculations were run with Gaussian 16,^39^ with default grids and convergence criteria. Conformational searches and the optimizations of the conformers obtained were performed as previously described.^40^ Final optimizations were run at the ωB97X-D/6-311+G(d,p) level, including the Solvation Model based on Density (SMD) for CH_3_CN. TD-DFT calculations were run with several functionals (CAM-B3LYP, B3LYP, BH&HLYP, M06-2X, and ωB97X-D) and def2-TZVP basis set, including a polarizable continuum solvent model (PCM) for CH_3_CN. Average ECD spectra were computed as previously described.^40^ All conformers with a population >1% at 300 K were considered; these amounted to two conformers for compound **1**, differing in the orientation of the ester moiety. ECD spectra were generated using the program SpecDis,^41^ as previously described.^42^

### Leaf Puncture Assay

Holm oak (*Q. ilex* L.) and tomato (*L. esculentum* L.) young leaves were used for this assay. Fungal culture filtrates, organic extracts, and each compound (**1**–**4**) were assayed at 1.0 mg/mL following the procedure previously described.^40^ Each treatment was repeated 3 times, and the leaves were scored for symptoms after 7 days. The phytotoxicity was expressed using a visual scale from 0 (no symptoms) to 4 (wide necrosis up to 1 cm in diameter). The toxic effects of compounds **1**–**4** were observed for up to 10 days.

### Tomato Cutting Assay

Tomato cuttings were taken from 21 day old seedlings of the fungal culture filtrates, and its organic extracts and chromatographic fractions were assayed at 1 mg/mL following the procedure previously described.^40^ Symptoms were evaluated visually up to 7 days, and the phytotoxicity was expressed with the same scale reported above.

## Supporting Information Available

Selection of 1D and 2D ^1^H and ^13^C NMR, HRESIMS, IR, and UV spectra (PDF)
