# Supplementary material for: Rabenchromenone and Rabenzophenone, Phytotoxic Tetrasubstituted Chromenone and Hexasubstituted Benzophenone Constituents Produced by the Oak-Decline-Associated Fungus Fimetariella rabenhorstii
Source: J Nat Prod. 2020 Jan 22;83(2):447–52. doi: 10.1021/acs.jnatprod.9b01017 (PMC7993755; doi:10.1021/acs.jnatprod.9b01017)
Supplement: Supplementary file 1 — np9b01017_si_001.pdf [file np9b01017_si_001.pdf]

SUPPORTING INFORMATION

Rabenchromenone and Rabenzophenone, Phytotoxic Tetrasubstituted Chromenone and Hexasubstituted Benzophenone Constituents Produced by the Oak Decline Associated Fungus *Fimetariella rabenhorstii*

Samaneh Bashiri, † Jafar Abdollahzadeh, † Roberta Di Lecce, ‡ Daniela Alioto,± Marcin Górecki,§,⊥ Gennaro Pescitelli,§ Marco Masi,*, ‡ and Antonio Evidente ‡

† Department of Plant Protection, Faculty of Agriculture, University of Kurdistan, Pasdaran St., P.O. Box 416, Sanandaj, Iran

‡ Dipartimento di Scienze Chimiche, Università di Napoli Federico II, Complesso Universitario Monte S. Angelo, Via Cintia 4, 80126 Napoli, Italy

±Dipartimento di Agraria, Università degli Studi di Napoli Federico II, Via Università 100, 80055 Portici, Italy

§Dipartimento di Chimica e Chimica Industriale, Università di Pisa, Via Moruzzi 13, 56124 Pisa, Italy

⊥Institute of Organic Chemistry, Polish Academy of Sciences, Kasprzaka 44/52 St., 01-224Warsaw, Poland

Supporting Information List

Page 3: **Spectra 1**. ^{1}H NMR spectrum of Rabenchromenone (**1**) ($CDCl_3$, 400 MHz).

Page 3: **Spectra 2**. ^{13}C NMR spectrum of Rabenchromenone (**1**) ($CDCl_3$, 100 MHz).

Page 4: **Spectra 3**. HSQC spectrum of Rabenchromenone (**1**) ($CDCl_3$, 400/100 MHz).

Page 4: **Spectra 4**. HMBC spectrum of Rabenchromenone (**1**) ($CDCl_3$, 400/100 MHz).

Page 5: **Spectra 5**. COSY spectrum of Rabenchromenone (**1**) ($CDCl_3$, 400 MHz).

Page 5: **Spectra 6.** ^{1}H NMR spectrum of Rabenzophenone (**2**) ($CDCl_3$, 400 MHz).

Page 6: **Spectra 7.** ^{13}C NMR spectrum of Rabenzophenone (**2**) ($CDCl_3$, 100 MHz).

Page 6: **Spectra 8.** HSQC spectrum of Rabenzophenone (**2**) ($CDCl_3$, 400/100 MHz).

Page 7: **Spectra 9.** HMBC spectrum of Rabenzophenone (**2**) ($CDCl_3$, 400/100 MHz).

Page 7: **Spectra 10.** COSY spectrum of Rabenzophenone (**2**) ($CDCl_3$, 400 MHz).

Page 8: **Figure S1.** ECD spectrum of (*R*)-**1** calculated with four different functionals and def2-TZVP basis set including PCM solvent model for acetonitrile, using input structures optimized at ωB97X-D/6-311+G(d,p)/SMD level, Boltzmann averaged over 2 conformers at 300 K. Calculated spectra were obtained as sums of Gaussian bands with 0.3 eV exponential half-width; BH&HLYP spectrum was red-shifted by 5 nm. The ECD spectra are not scaled.

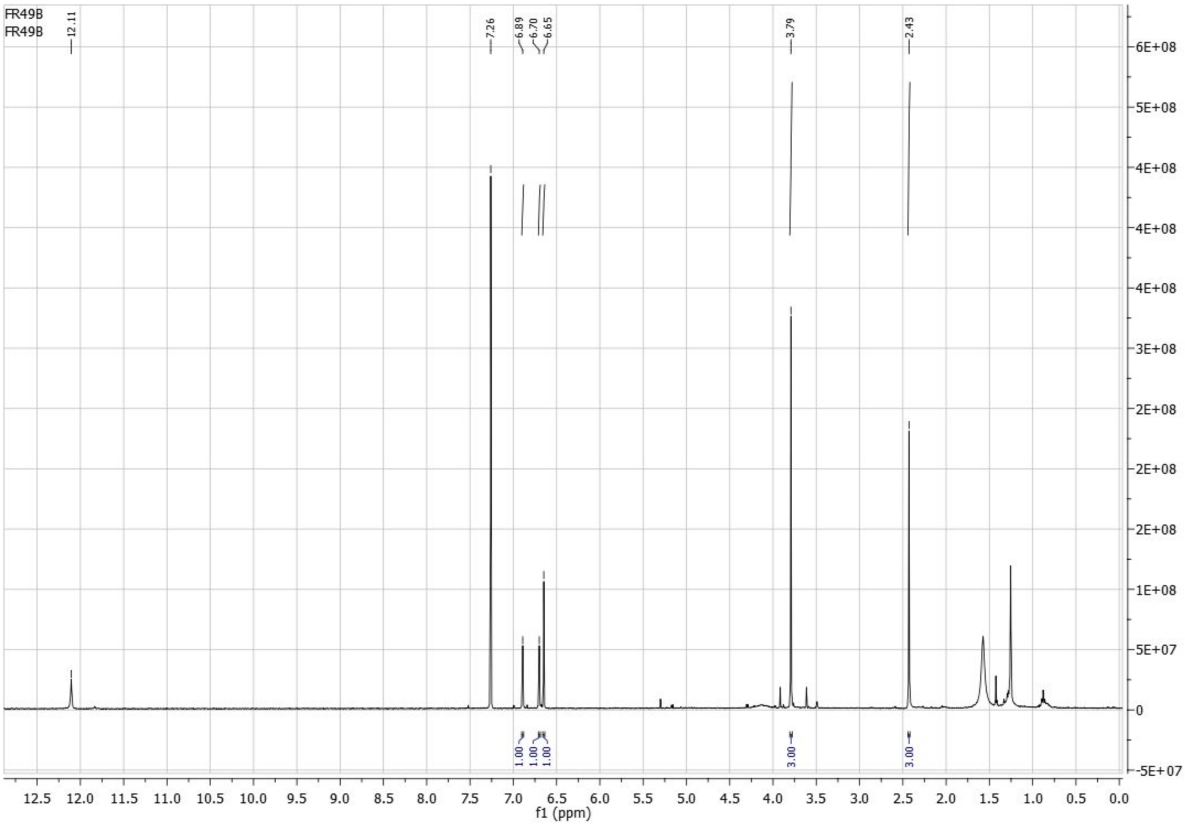

Spectra 1. ^{1}H NMR spectrum of Rabenchromenone (**1**) ($CDCl_3$, 400 MHz).

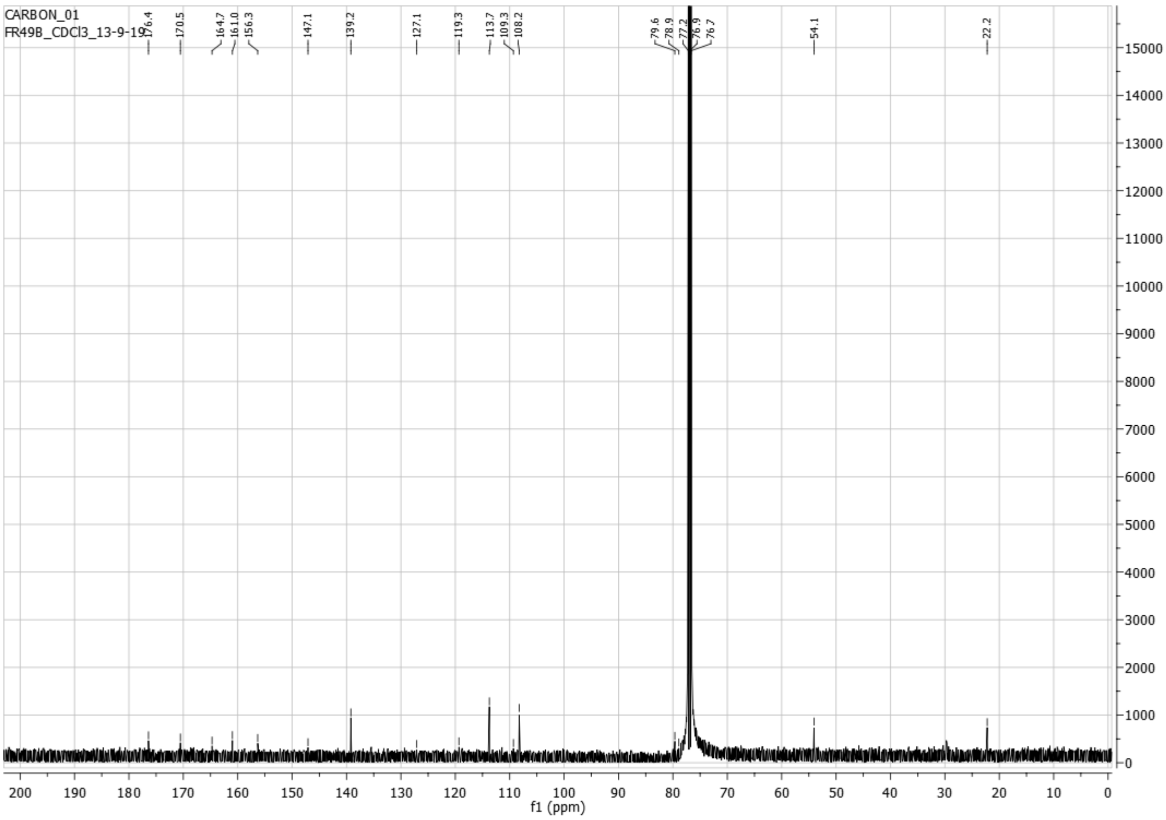

Spectra 2. ^{13}C NMR spectrum of Rabenchromenone (**1**) ($CDCl_3$, 100 MHz).

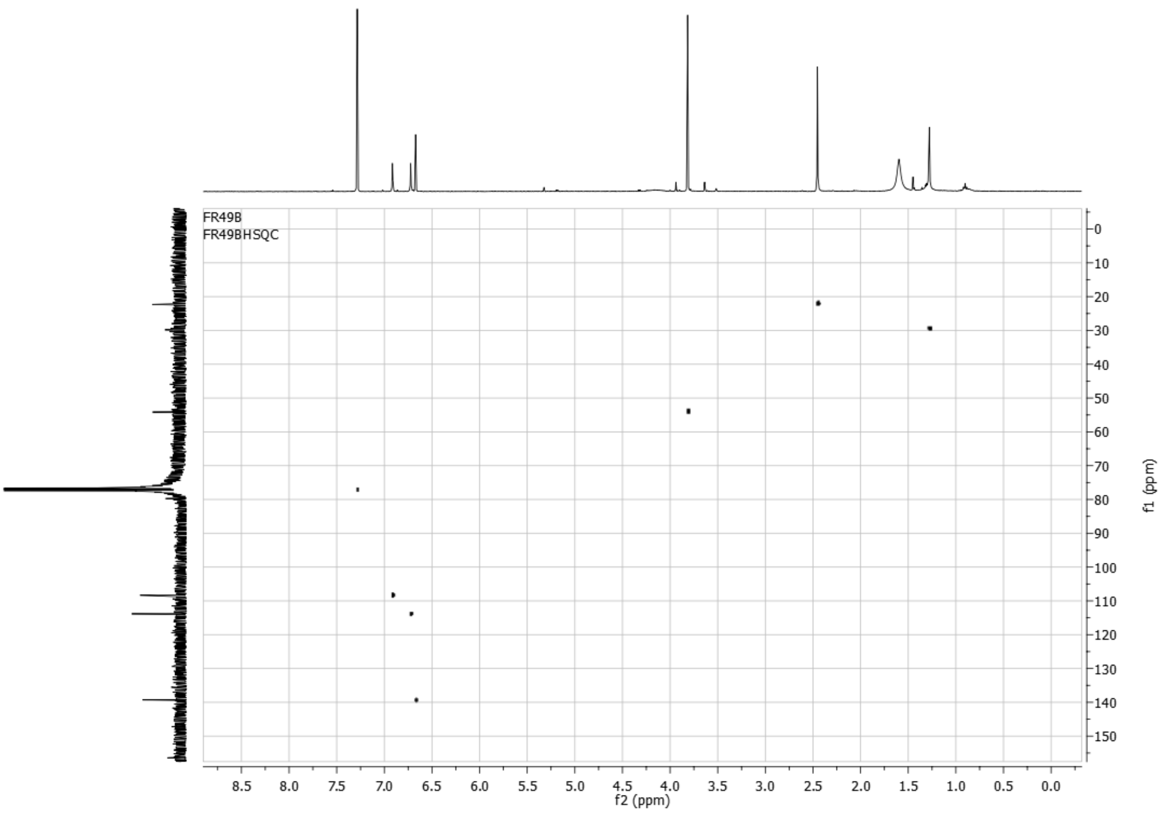

Spectra 3. HSQC spectrum of Rabenchromenone (**1**) ($CDCl_3$, 400/100 MHz).

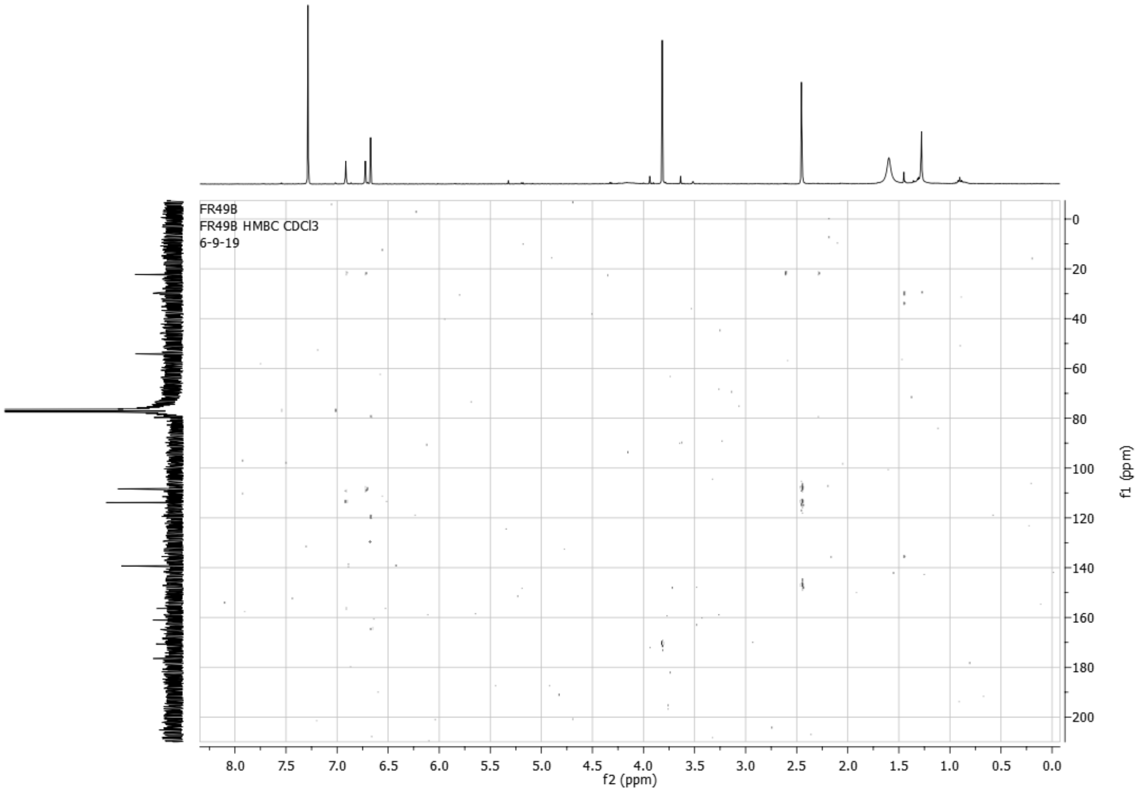

Spectra 4. HMBC spectrum of Rabenchromenone (**1**) ($CDCl_3$, 400/100 MHz).

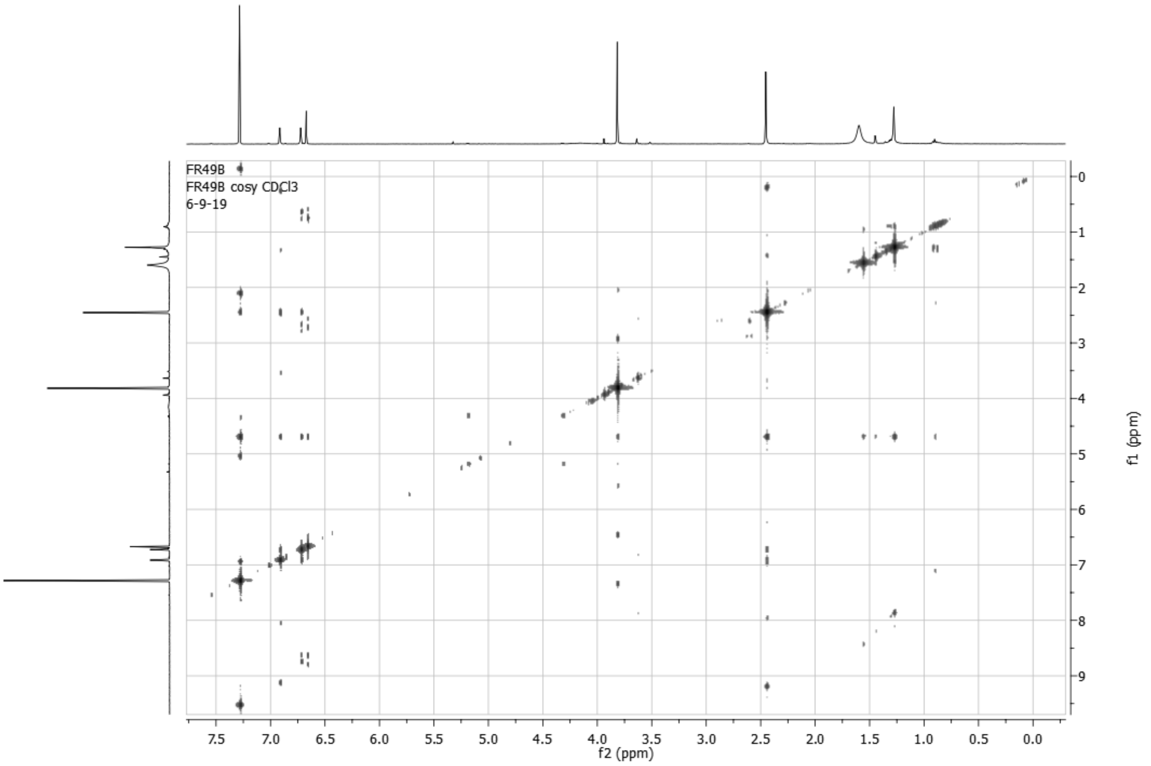

Spectra 5. COSY spectrum of Rabenchromenone (**1**) ($CDCl_3$, 400 MHz).

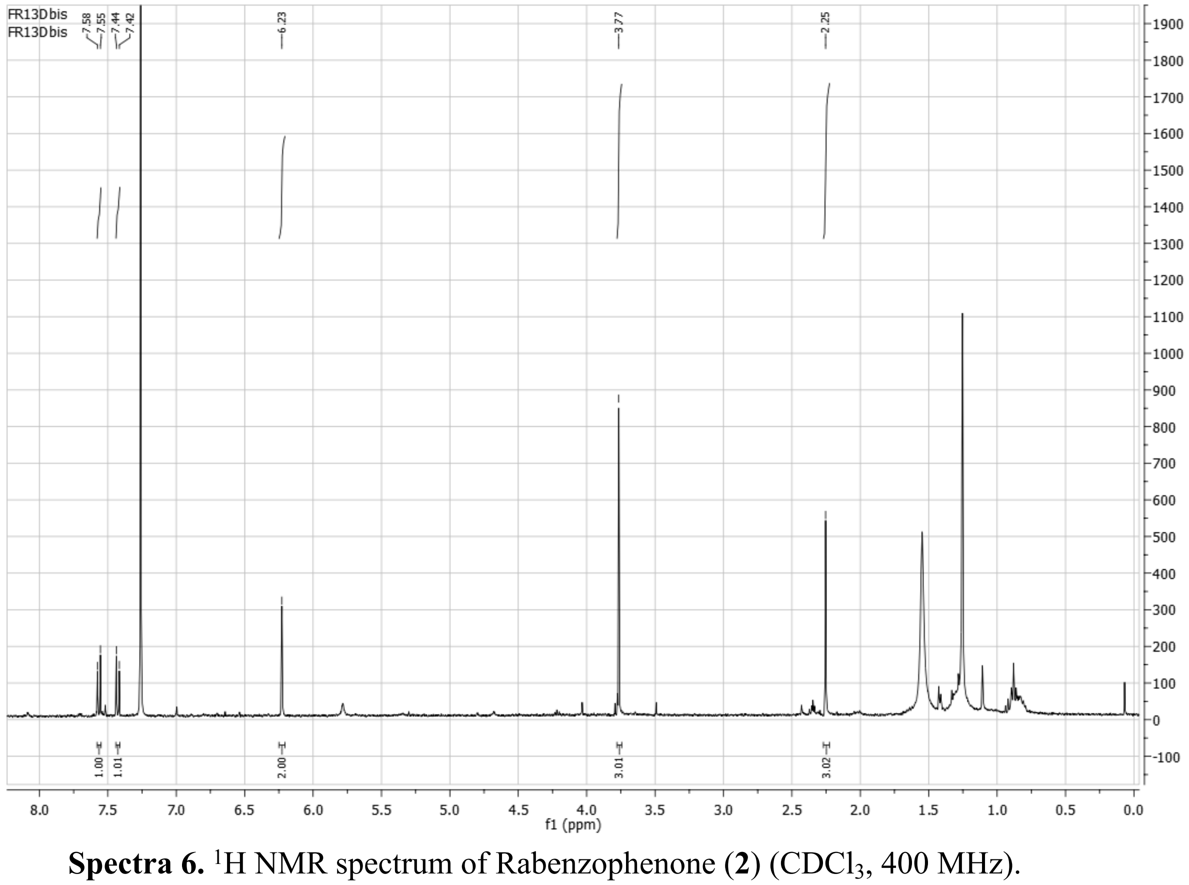

Spectra 6. ^{1}H NMR spectrum of Rabenzophenone (**2**) ($CDCl_3$, 400 MHz).

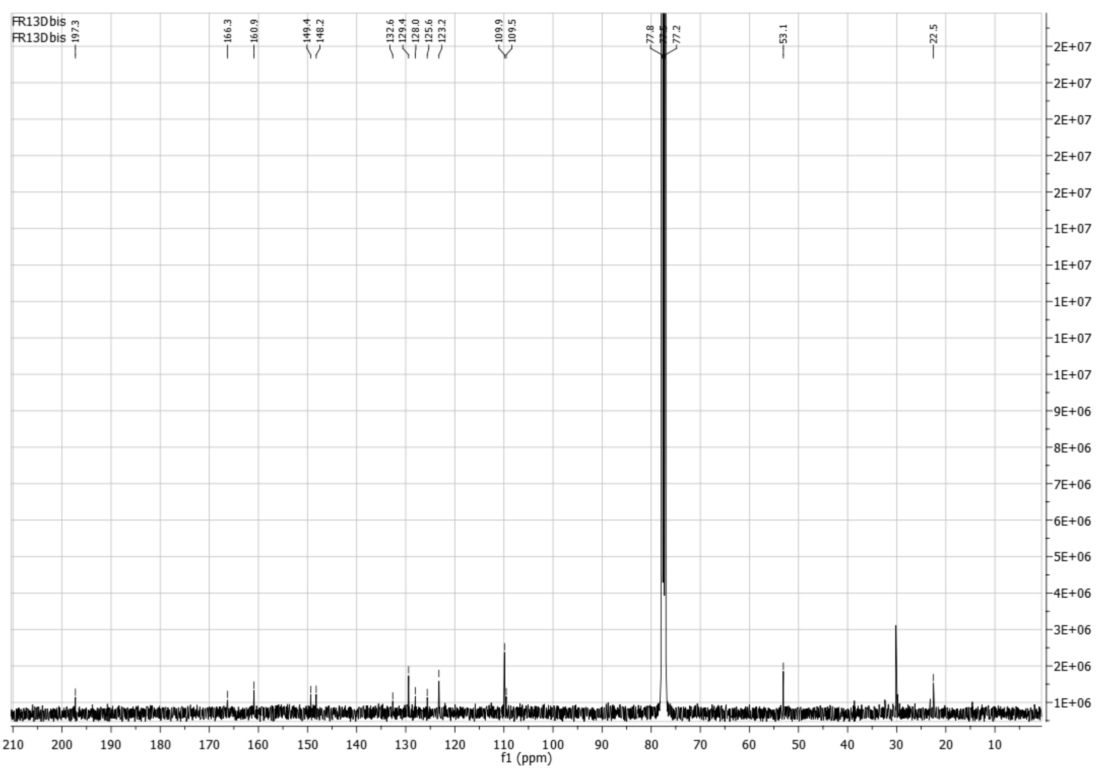

Spectra 7. ^{13}C NMR spectrum of Rabenzophenone (**2**) ($CDCl_3$, 100 MHz).

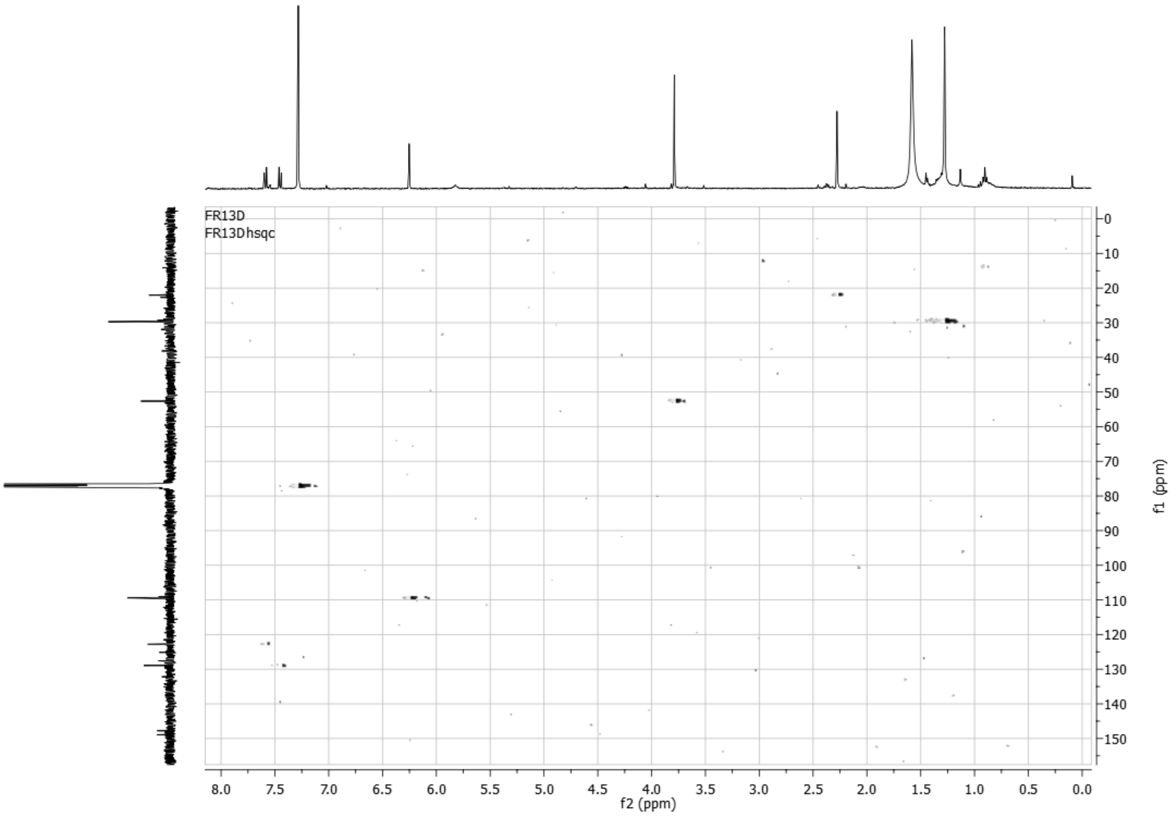

Spectra 8. HSQC spectrum of Rabenzophenone (**2**) ($CDCl_3$, 400/100 MHz).

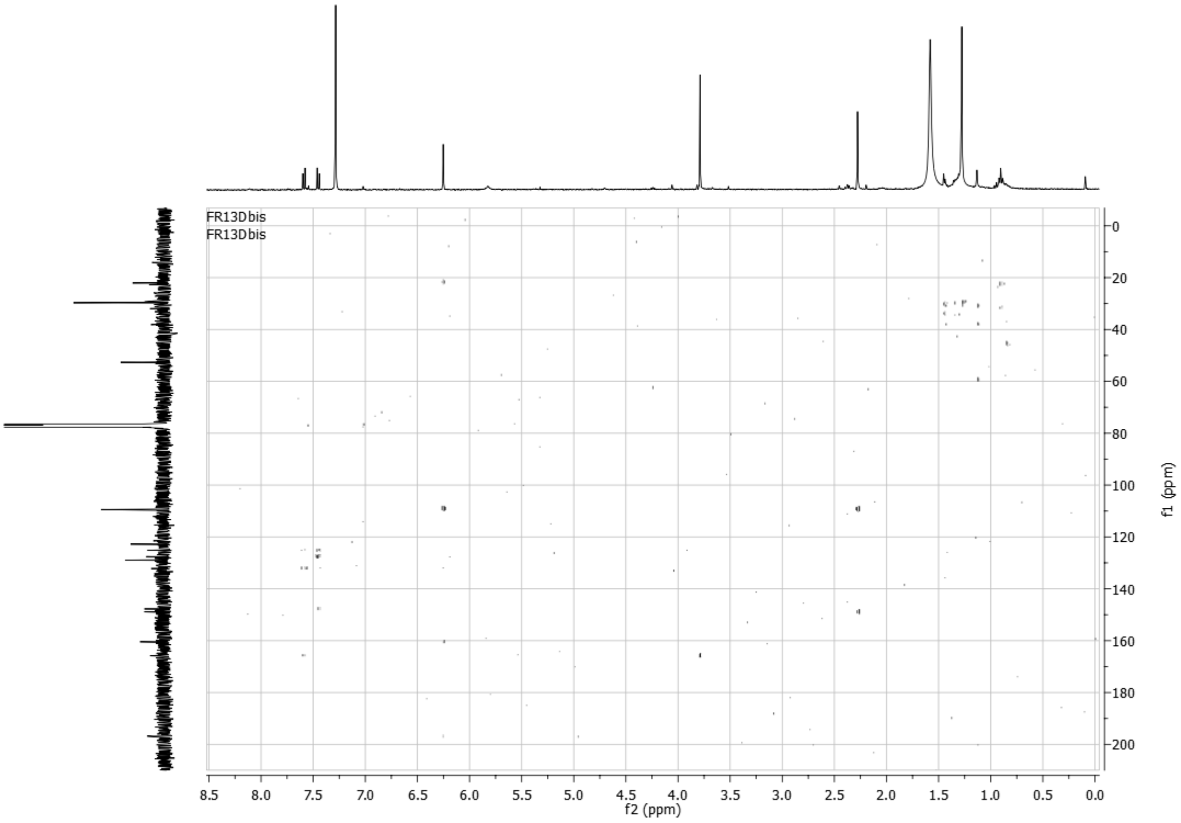

Spectra 9. HMBC spectrum of Rabenzophenone (**2**) ($CDCl_3$, 400/100 MHz).

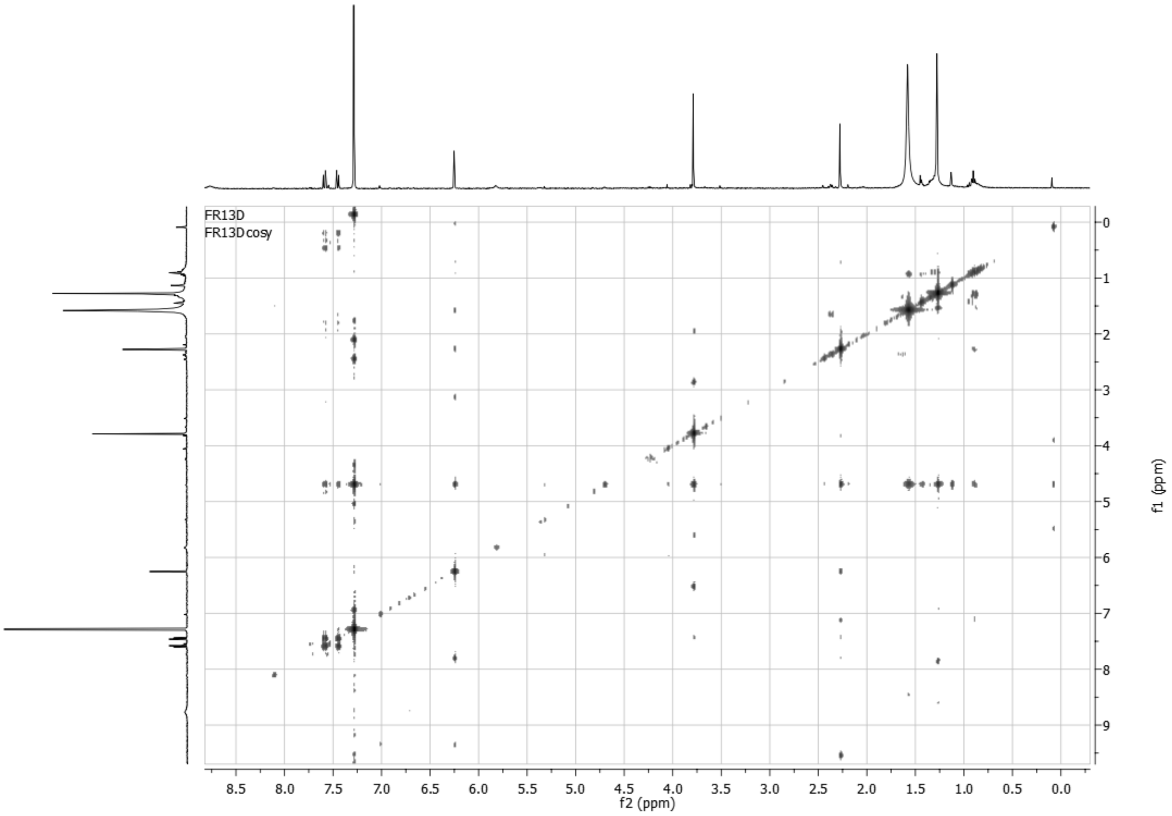

Spectra 10. COSY spectrum of Rabenzophenone (**2**) ($CDCl_3$, 400 MHz).

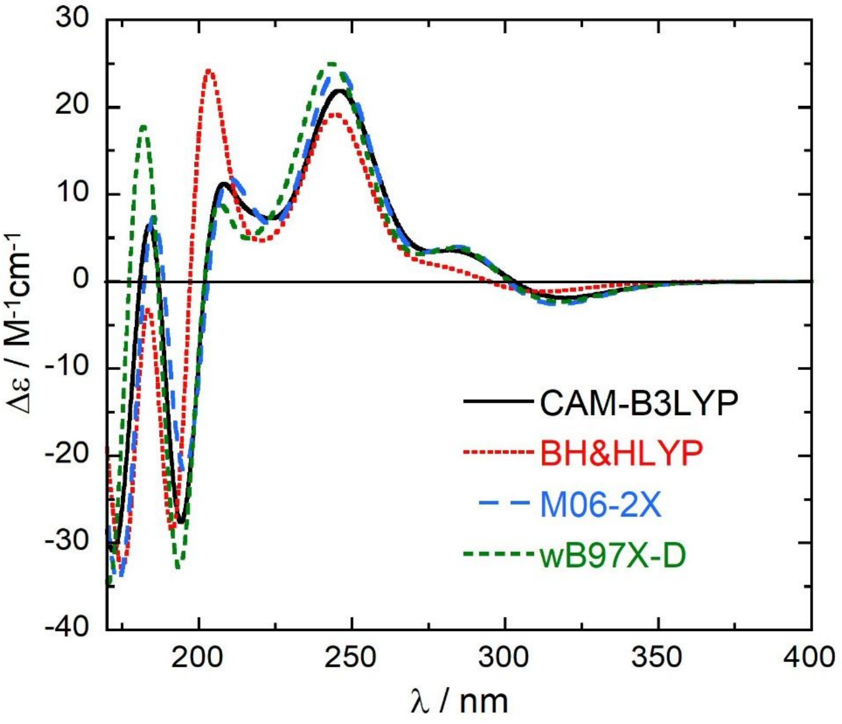

Figure S1. ECD spectrum of (*R*)-**1** calculated with four different functionals and def2-TZVP basis set including PCM solvent model for acetonitrile, using input structures optimized at ωB97X-D/6-311+G(d,p)/SMD level, Boltzmann averaged over 2 conformers at 300 K. Calculated spectra were obtained as sums of Gaussian bands with 0.3 eV exponential half-width; BH&HLYP spectrum was red-shifted by 5 nm. The ECD spectra are not scaled.
